# Nucleus accumbens DRD2 receptor agonism attenuates escape behavior

**DOI:** 10.3389/fnins.2026.1881674

**Published:** 2026-07-13

**Authors:** Bridgitte E. Côté, Elaine M. Grafelman, Lisa Moster, Kara Zimolzak, Hannah E. Hix, Matthew Cooper, G. Nino Padula, Daniel S. Wheeler, Matthew C. Hearing, John R. Mantsch, Robert A. Wheeler

**Affiliations:** 1 Department of Biomedical Sciences, Marquette University, Milwaukee, WI, United States; 2 Department of Pharmacology and Toxicology, Medical College of Wisconsin, Milwaukee, WI, United States

**Keywords:** aversion, dopamine, DRD2, escape, medium spiny neurons, negative reinforcement, nucleus accumbens, quinpirole

## Abstract

Animals learn to approach and escape stimuli in their environment, in part through the representation of rewarding or aversive outcomes in the nucleus accumbens (NAc). The regulation of reward motivation in the NAc by dopamine signaling at DRD1 and DRD2 receptors has been the subject of extensive study. However, the process by which aversive stimuli are signaled within this system to promote motivated escape behavior is less well characterized. Conventional wisdom posits that rewarding and aversive stimuli ultimately affect DRD1 or DRD2-receptor expressing medium spiny neurons (MSNs) in an opposing manner to differentially modulate motivated behavior. However, recent studies have challenged this view and demonstrate the need to better characterize the processes that mediate aversion learning. To determine if DRD2 dopamine receptor activation disrupts escape behavior, 21 male and female Sprague Dawley rats were treated with an intra-NAc core DRD2 receptor agonist, quinpirole, while escape behavior was negatively reinforced by the termination of aversive white noise. This treatment attenuated escape, a result that is consistent with the view that aversion-induced reductions in dopamine promote escape behavior through decreased DRD2 receptor signaling in the NAc, and potential disinhibition of an aversion-sensitive striatal output circuit.

## Introduction

The learning of instrumental responses to pursue rewarding stimuli and escape aversive stimuli is essential for an animal to effectively navigate the environment. Thus, reward learning has been the subject of extensive study, which has revealed a sophisticated system that guides appropriate behavioral action selection (Howland et al., 2022). The processing of aversive stimuli that guides escape and avoidance involves this system as well, but this process is not as well characterized (Verharen et al., 2020). In fact, recent studies contradict prior conventional wisdom positing that rewarding and aversive stimuli engage this system in an opposing manner (Vieitas-Gaspar et al., 2025), necessitating further characterization of the processes that mediate aversion learning.

While it is well known that aversive experiences have a profound effect on behavior, far less is known about how those experiences are represented in the brain areas that modulate ongoing behavior. The nucleus accumbens (NAc) contributes to this by integrating a range of cortical and limbic information about rewarding and aversive experiences and then mediating behavior through projections to motor output pathways. It is well established that rewarding stimuli promote behavior, in part, through increases in NAc dopamine (Wise, 2004), but NAc dopamine is also involved in aversion-mediated behavior. Our lab and others have characterized a rapid reduction in NAc dopamine in response to a range of aversive stimuli including loud (90db) white noise (Elzelingen et al., 2022; Goedhoop et al., 2022; Grafelman et al., 2025), bitter quinine (Roitman et al., 2008; Twining et al., 2015), and foot shock (Stelly et al., 2019). This signal is associated with the engagement of aversion-related behaviors including promoting escape and drug taking (Grafelman et al., 2025; Mahler et al., 2019; Oleson et al., 2012).

A central mechanism by which NAc dopamine mediates these responses is through its actions at dopamine-sensitive medium spiny neurons (MSNs) that are both the projection neurons and the primary neuron type in the region. It has been proposed that reduced dopamine signaling may influence aversion processing through reduced occupancy of the inhibitory (Gi) DRD2 receptor, thus disinhibiting activity of the DRD2 receptor-expressing MSNs (D2 MSNs). This could align with *in vivo* electrophysiology recordings that characterize predominantly increased activity in NAc MSNs in response to aversive stimuli (Roitman et al., 2005; Roitman et al., 2010; Wheeler et al., 2015). Previously observed phasic activity of MSNs to aversive stimuli may reflect such disinhibition and support a proposed model where DRD2 receptor-expressing MSNs signal aversion and suppress goal-directed behavior. The results of behavioral experiments have supported this model, demonstrating that D2 MSN activity is necessary for punishment learning (Robble et al., 2020), and conditioned place aversion (Danjo et al., 2014). Stimulated D2 MSN activity can also induce transient punishment (Cole et al., 2018; Lobo et al., 2010), and risk-aversion (Zalocusky et al., 2016). However, there is also evidence that conflicts with this view, demonstrating that stimulated D2 MSN activity can increase motivation for rewards (Soares-Cunha et al., 2018; Soares-Cunha et al., 2020; Soares-Cunha et al., 2022). Therefore, the manner by which DRD2 receptor activity supports both avoidance and reward motivation is unclear.

We hypothesize that altered DRD2 signaling by aversion-induced reductions in dopamine is necessary for appropriate escape behavior. To test this, we pharmacologically activated DRD2 receptors during negative reinforcement learning. In this negative reinforcement design, rats learned to escape an aversive white noise stimulus by engaging in an operant response. The results indicate that maintaining activity of DRD2 dopamine receptors during the experience of an aversive stimulus disrupts the acquisition of escape behavior.

## Materials and methods

### Subjects

Twenty-one (11 female, 10 male) Sprague Dawley Rats (Envigo) were housed in an AAALAC-accredited vivarium at Marquette University. Animals were housed in a temperature-controlled room, under a reverse 12:12 light/dark cycle (light onset at 19:00 CST). Food and water were available ad libitum. All animal housing conditions and procedures are approved by Marquette University’s IACUC.

### Pharmacology

#### Surgery

All surgical procedures were conducted under isoflurane anesthesia (induction at 4% then maintained at 2–2.5%). Rats (205-375 g) received stereotaxic intracranial surgery to implant a guide canula (11 mm) into the NAc core (10° angle: AP + 1.3, ML +/−2.4, DV –6.5) for microinjections. The headcap was secured to the skull using 4 skull screws and dental cement (Ortho-Jet). For 7 days post-surgery, rats were monitored and administered the analgesic, meloxicam (first 2 days and then as needed).

#### Microinfusions

Rats received a microinfusion of either sterile saline or quinpirole 15 min before negative reinforcement. On the morning of each treatment day, an aliquot of the dopamine DRD2-like receptor agonist, quinpirole mixed with sterile saline (2 μg/1 μL, stored at −80°) was thawed for testing. Sterile saline or quinpirole (1.0 μg/60s) was bilaterally injected into the NAc core using microinjectors that extend 0.5 mm from the end of the guide cannula (Robble et al., 2020; Porter-Stransky et al., 2013). Microinjectors were connected to a 10 μL Hamilton syringe (Reno, NV, United States) in a microinfusion pump (KdScientific) by polyethylene (PE) tubing filled with sterile milliQ water. The infusion volume of 0.5 μL per side was delivered over 60 s and microinjectors were left in place for 2 min after each injection to allow for diffusion. PE tubing, Hamilton syringe, microinjectors and all other supplies used for infusions were flushed with 200 proof ethanol followed by sterile milliQ water after each testing day.

### Behavioral paradigm

#### Negative reinforcement

Rats began behavioral testing with an autoshaping design for negative reinforcement. Negative reinforcement took place in an operant chamber (MED Associates) equipped with two nose poke ports (MED Associates) in the bottom left and right sides of one of the chamber walls. The opposite wall was equipped with two speakers (MED Associates) on the top left and right sides. There was also a perforated metal sheet used to block one half of the operant chamber to confine the available space to the side with the nose poke ports and encourage rats to interact with them. At the start of the session, an aversive 90db white noise (WN) turned on. This noise could be terminated for 10 s by an entry into one of two nose pokes (active, counterbalanced). White noise remained on throughout the session, except during the 10 s intervals after each active nose poke. The sessions lasted 90 min.

Initial pilot testing (using a separate cohort of rats) indicated that starting vehicle infusions during the first day of negative reinforcement training significantly impaired acquisition. Therefore, rats began initial negative reinforcement training for at least 3 days or until meeting criteria; 3 consecutive days of 50 or more white noise terminations (baseline). After meeting criteria, rats underwent four treatment days where they received bilateral 0.5 μL microinfusions of either quinpirole (*n* = 12, 5F) or saline (*n* = 9, 5F) 15 min before the start of behavioral testing. After the four treatment days, rats continued negative reinforcement for an additional 3 days to capture lasting effects of quinpirole (washout).

#### Histology

**Figure 1:**
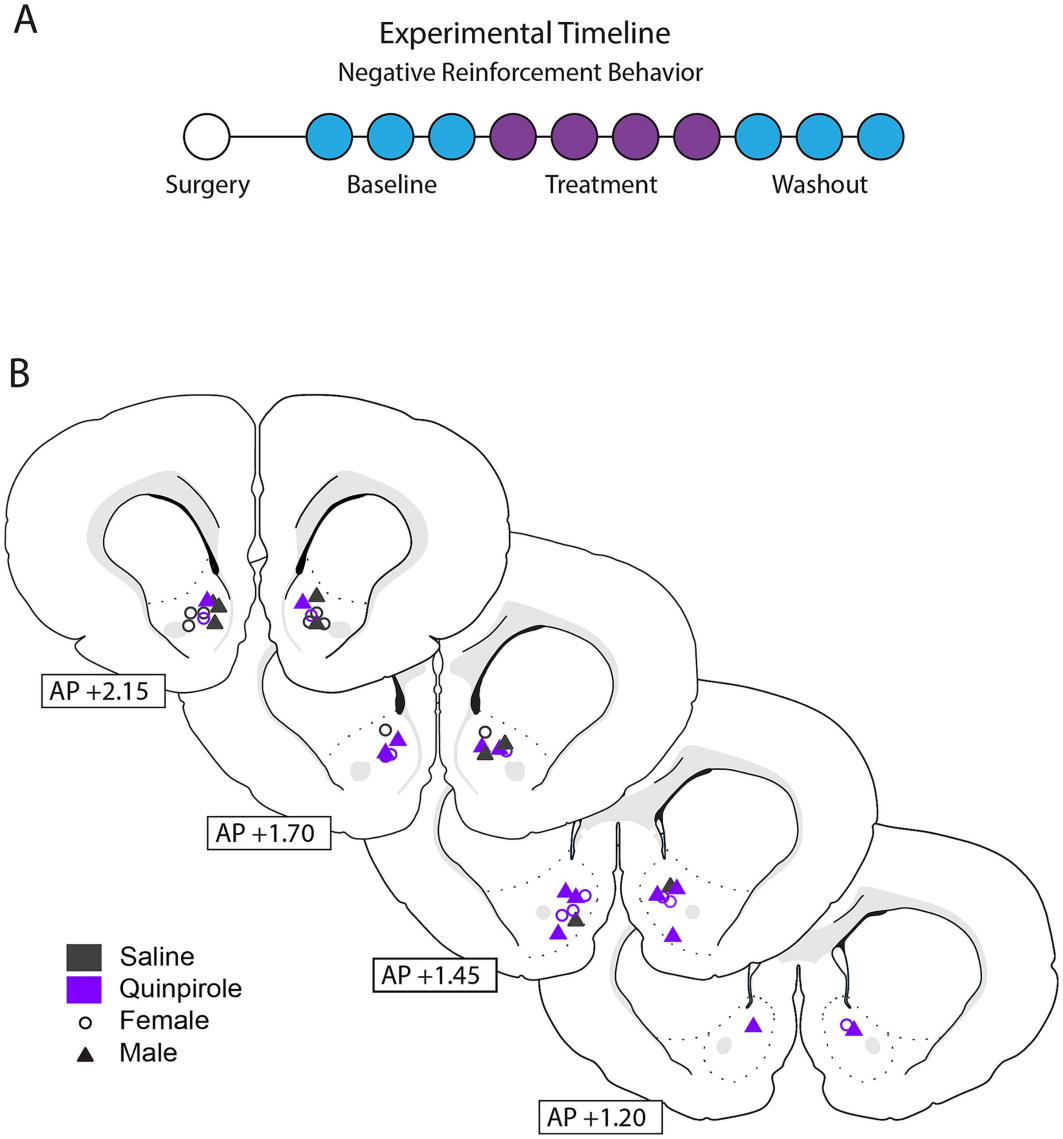
Experimental design. **(A)** Experimental timeline and **(B)** histological verification of NAc cannula placements. Placements were verified to be in the NAc core or border between the NAc core and shell. Treatment group (symbol color): Quinpirole (purple); Vehicle (gray); Sex (Symbol): Male (triangle); Female (circle).

Upon completion of each experiment, rats were euthanized with a lethal injection of urethane (2,000 mg/Kg; intraperitoneal) and transcardially perfused (saline and 4% paraformaldehyde). After perfusions, brains were harvested and stored in 4% paraformaldehyde for 24 h and transferred to 0.05 M phosphate buffered saline. Using a vibratome, 50-micron slices were taken of the nucleus accumbens. Slices were then visualized using a Keyence microscope at 10x magnification to evaluate cannula placement (Figure 1B).

### Data analysis

Group and sex differences in all measures were examined using commercially available software (SPSS). Each dataset was analyzed with a mixed factorial ANOVA. To test the effect of quinpirole on escape behavior, the 3 phases of the experiments were compared: Baseline, days −3 to −1, Treatment: days 2–4, and Washout: days 5–7. Day 1 was omitted from the analyses to allow for appropriate between and within comparisons across phases. To determine the effect of quinpirole as a change from baseline, 2 phases of the experiments were compared: Treatment: days 2–4, and Washout: days 5–7. Planned linear contrasts (i.e., planned comparisons) were conducted, where an *a priori* hypothesis dictated, based on the results of the ANOVA. The *a priori* hypothesis that guided comparisons was that aversive white noise promoted escape. Sex was used as a variable in all analyses. However, a *post hoc* power analysis using G*Power software (Version 3.1.9.7; Universitat Kiel, Germany) indicated that analyses were underpowered to detect sex differences (1-*β* = 0.40–0.70).

## Results

### Pre-treatment baseline

**Figure 2:**
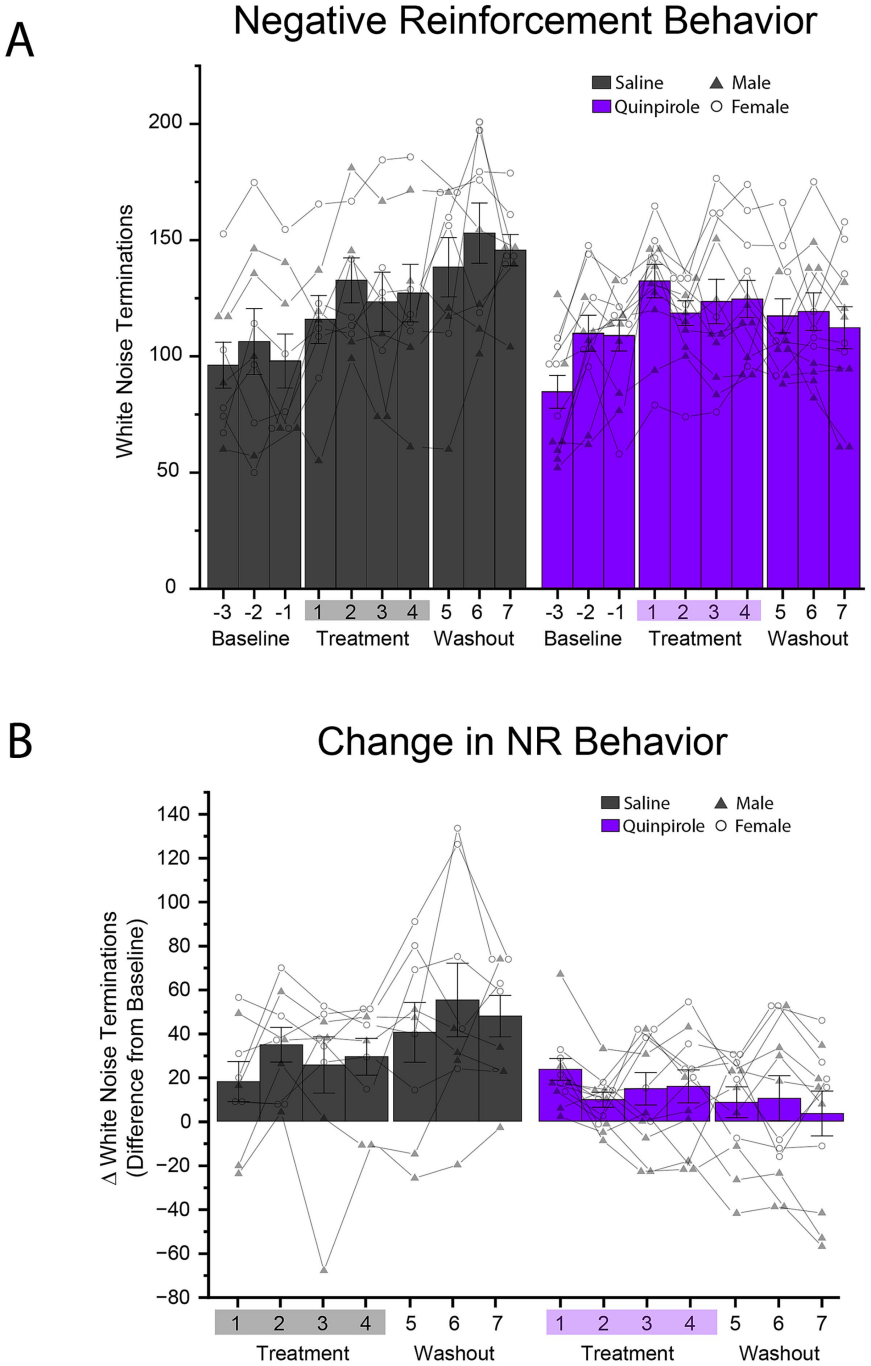
Quinpirole attenuated escape behavior. **(A)** Mean number of responses to terminate white noise within a 90-minute session for rats receiving intra NAc-core infusions of either saline or quinpirole. Quinpirole-treated rats performed fewer white noise terminations than their saline-treated counterparts, *f*(2,34) = 3.60, *p* = 0.038. **(B)** Mean difference in the number of responses to terminate white noise compared with the day immediately prior to treatment. Saline-treated rats continued to perform more terminations across treatment days, while quinpirole-treated rats decreased responding, *f*(1,17) = 4.479, *p* = 0.049. Overall, female rats increased terminations more than males, *f*(1,17) = 6.795, 331 *p* = 0.018. Treatment group (symbol color): Quinpirole (purple); Vehicle (gray); Sex (Symbol): Male 332 (triangle); Female (circle). Highlighted days indicate treatment period for saline (gray) or quinpirole (purple) infusions.

Rats were trained in a negative reinforcement autoshaping design for at least 3 days prior to microinfusions (Figure 1A). A Sex (2) × Drug (2) × Day (3) repeated measures ANOVA revealed a main effect of day, indicating that the number of white noise terminations increased over the three training days as rats acquired the escape behavior, *f*(2,38)=9.066, *p* < 0.001, (Figure 2, days −3 to −1). There were no significant differences between experimental groups prior to testing.

### Effects of quinpirole on negative reinforcement

#### White noise terminations

To test the effect of DRD2 receptor activation on negative reinforcement behavior, rats received microinfusions of either quinpirole or saline into the NAc before daily negative reinforcement testing. A significant Phase x Drug interaction revealed that quinpirole-treated rats performed fewer white noise terminations than their saline counterparts *f*(2,34)= 3.60, *p* = 0.038. Based on this, separate analyses of each Phase indicated that the groups did not differ during the baseline phase (days –3 to –1), *f*(1,19)= 0.007, *p* > =0.05), but a significant Drug × Day interaction during the treatment period (days 1 to 4) indicated that quinpirole reduced escape behavior, *f*(3,63) = 2.90, *p* = 0.042. A main effect of Drug in the washout phase (days 5–7), demonstrated that quinpirole treated rats continued to perform fewer escape attempts, *f*(1,19)= 7.01, *p* < 0.01, Figure 2A. There was no significant main effect or interaction involving sex.

#### Change from baseline

Total escape attempts were also transformed to analyze the difference in negatively reinforced behavior across days after the start of treatment. The number of white noise terminations from days 2–7 were subtracted from performance on the day before treatment (day −1). A significant Phase × Drug interaction indicated that saline-treated rats continued to perform more terminations across treatment days, while quinpirole-treated rats maintained or decreased responding, *f*(1,17)= 4.479, *p* = 0.049. Based on this, separate analyses of each Phase indicated that there was a significant Drug × Day interaction during the treatment period, *f*(3,51)= 3.26, *p* = 0.029. There was no significant interaction in the washout period, but there was a significant main effect of Drug, *f*(1,19)= 8.38, *p* < 0.01, indicating that quinpirole treated rats continued to perform fewer escape attempts, Figure 2B. A significant main effect of sex was also observed in this analysis, indicating that female rats performed more terminations than their male counterparts regardless of drug condition, *f*(1,17)= 6.795, *p* = 0.018.

#### Inactive nosepoke responding

**Figure 3:**
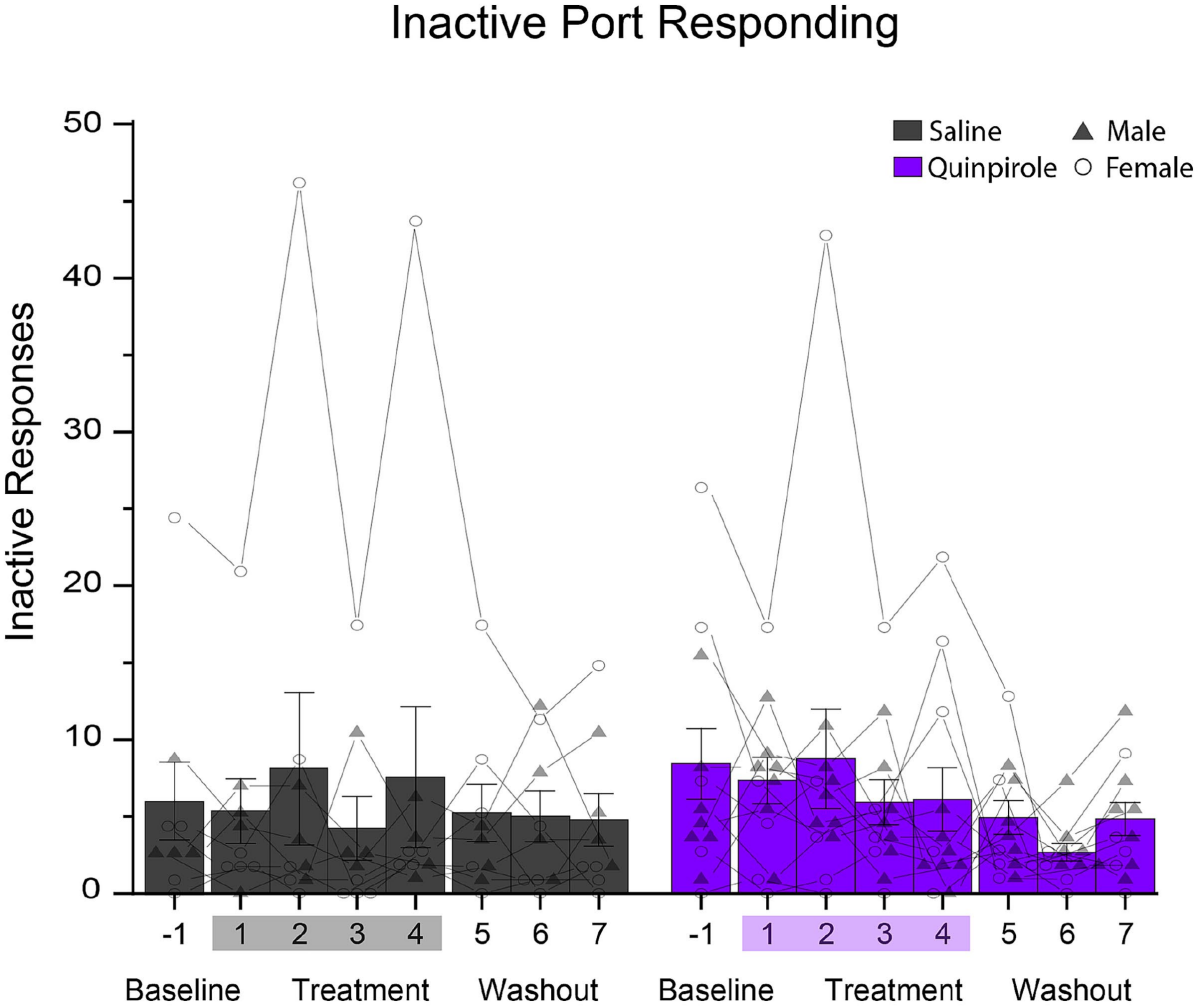
Quinpirole did not affect non-goal directed behavior. Mean number of response in the inactive port within a 90-minute session for rats receiving intra NAc-core infusions of either saline or quinpirole. There were no significant between or within subjects differences in inactive responses, (*f*(1,17) = 0.58, *p* = 0.812). Treatment group (symbol color): Quinpirole (purple); Vehicle (gray); Sex (Symbol): Male (triangle); Female (circle). Highlighted days indicate treatment period for saline (gray) or quinpirole (purple) infusions.

To determine if quinpirole administration affected non-goal directed behavior, inactive nosepoke responding was measured throughout training. There were no significant differences in inactive responding between treatment groups or sexes, *f*(1,17)= 0.58, *p* = 0.812, Figure 3. The general low level of responding is not ideal evidence of unimpaired motor activity. However, if quinpirole were to acutely suppress locomotion, this would be apparent in active responses on the on the first treatment day. We did not observe such an effect (Figure 2). In addition, prior work from our lab and others have used the same quinpirole microinfusion procedure and not observed suppressed behavior (Robble et al., 2020; Porter-Stransky et al., 2013) suggesting that the change in escape behavior is driven by a change in goal-directed behavior rather than locomotor suppression.

#### Average latency

**Figure 4:**
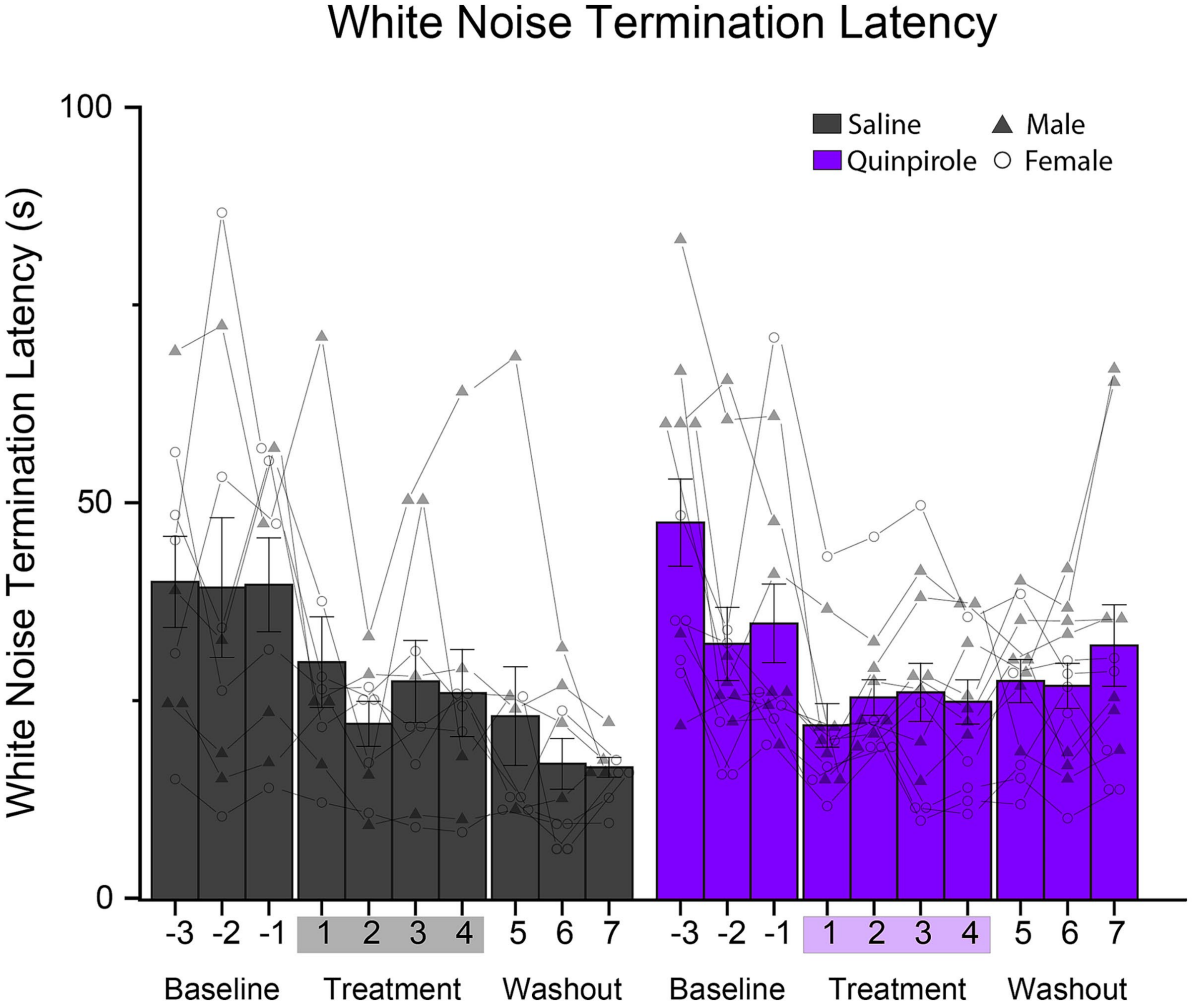
Quinpirole increased latency to escape. Average latency to terminate white noise for rats receiving intra NAc-core infusions of either saline or quinpirole. The effect of quinpirole on latency 11 to escape approached significance, *f*(4,68) = 2.165, *p* = 0.082. Treatment group (symbol color): Quinpirole (purple); Vehicle (gray); Sex (Symbol): Male (triangle); Female (circle). Highlighted days indicate treatment period for saline (gray) or quinpirole (purple) infusions.

In addition to measuring the amount of white noise terminations, the latency between each instance of white noise onset and the termination was also analyzed. While this measure is related to the number of terminations (in a fixed duration training session, more terminations would necessarily be associated with shorter average latencies), it is not directly dependent on the number of noise terminations. Consistent with prior analyses, a Phase × Day × Drug interaction approached significance, *f*(4,68) = 2.165, *p* = 0.082, Figure 4. No other main effects or interactions involving Drug or Sex approached significance.

## Discussion

The current study tested the contribution of increased NAc DRD2 receptor activity to negative reinforcement learning. To test the role of increased DRD2 dopamine receptor activity in such behaviors, we administered the DRD2-like receptor agonist, quinpirole during negative reinforcement learning. Using a negative reinforcement task (Grafelman et al., 2025), we found that administration of quinpirole in the NAc reduced the number of responses to escape an aversive white noise.

Interestingly, this effect persisted even during the washout period (days 5–7). It is possible that this is due to an incomplete washout of quinpirole or altered dopamine transmission that has been observed after extended exposure to quinpirole. But to our knowledge, there is no evidence that a targeted delivery of low dose quinpirole would remain active (Whitaker and Lindstrom, 1987), or alter dopamine transmission at a comparable timescale (Henry et al., 1998; Escobar et al., 2015; Koeltzow et al., 2003). Rather, this lasting effect may reflect deficits in aversion learning and the related neural adaptations. The increasing trendline observed in the saline group in Figure 2 suggests that animals acquired escape behavior throughout the treatment period. Therefore, it is likely that quinpirole impaired the acquisition of this negatively reinforced behavior. More specifically, the saline group acquired an adaptive escape response through repeated associative learning trials, resulting in robust, experience-dependent potentiation. It is likely that quinpirole disrupted the experience of the aversive stimulus, the ability to learn from the aversive experience, or both. The result was a weaker association and conditioned escape response.

There is an extensive literature showing that DRD2 agonists (including quinpirole) can serve as a positive reinforcer (Caine et al., 2002; Caine and Koob, 1993; White et al., 1991) and facilitate instrumental responding for rewards (Collins and Woods, 2009; Ranaldi and Beninger, 1994; Schmidt et al., 2006; De Vries et al., 2002). However, less is known about how DRD2 agonists affect aversion learning. The present results are consistent with findings that DRD2 agonism impairs learning in aversion-mediated tasks. Prior work demonstrates that quinpirole prevents learned avoidance of an aversive outcome, or punishment learning (Robble et al., 2020; Porter-Stransky et al., 2013). The current study advances these studies by employing a task in which an aversive stimulus increases, rather than suppresses, motivated behavior. Together, these findings indicate that DRD2 receptor signaling has an important role in shaping an animal’s acquisition of escape response to aversive outcomes. Specifically, it suggests that reduced receptor activity is necessary for the acquisition of an adaptive escape response.

Interestingly, there are several studies that demonstrate that DRD2 agonists disrupt learning in paradigms that involve a rule change such as attentional set-shifting (Kurylo, 2004; Haluk and Floresco, 2009; Yawata et al., 2012), reflecting a deficit in cognitive flexibility. Conversely, optogenetic stimulation of D2 MSNs can enhance reversal learning (Wang et al., 2019). Because a rule change requires an animal to learn from a disappointing outcome when a former strategy fails, it is interesting to consider the possibility that DRD2-agonism impairs new rule learning in such tasks by preventing the aversive experience of such failures.

### A model of signaling during reward and punishment through opposing valence encoding

These findings align with a previously proposed model of the role of striatal signaling during reward and punishment learning (Robble et al., 2020). It is well established that dopamine in the NAc is released in response to rewarding stimuli (Wise, 2004). Additionally, our lab and others have found that aversive stimuli cause a rapid reduction in NAc dopamine, and that this reduction is an important regulator of aversion-mediated behavior (Grafelman et al., 2025; Roitman et al., 2008; Twining et al., 2015; Roitman et al., 2010; Robble et al., 2020; Wheeler et al., 2011) [but see (Kutlu et al., 2021)]. Due to the difference in the affinity of each dopamine receptor subtype, these dopamine spikes and troughs preferentially affect DRD1 and DRD2 receptor activity, respectively (Dreyer et al., 2010; Richfield et al., 1989). In the dorsal striatum, it is well characterized that this functionally distinguishes DRD1- and DRD2- expressing MSNs (D1 MSNs and D2 MSNs) as a part of the direct “go” and indirect “no-go” pathways that mediate movement. An opposing-valence model posits a parallel structure in the ventral striatum in which D1 MSN activity supports reward processing and D2 MSN activity supports aversion processing, functioning as a reward/aversion “go”/“no-go” pathways, respectively.

### The complex role of D1 and D2 MSN activity in reward and punishment

Recent research has revealed inadequacies in this simple model. Calcium imaging demonstrates that both D1 and D2 MSNs can be activated by aversive stimuli. Therefore, some have proposed that D1 and D2 MSN activity works in tandem to encode salience and learning, rather than opposing valence (Zachry et al., 2023). Some pharmacological, and more recent optogenetic manipulations have also found that D2 MSN activity can (using certain parameters) increase motivation, challenging an opposing-valence model (Soares-Cunha et al., 2018; Soares-Cunha et al., 2020; Domingues et al., 2025; Soares-Cunha et al., 2016). This view could also be used to interpret the present results; disrupted D2 MSN activity may attenuate behavior by suppressing associative learning, rather than aversion signaling. Although this model could not easily account for the faciliatory effects of DRD2 agonists on associative learning in positive reinforcement designs.

In addition to conflicting behavioral evidence, a growing body of work has revealed that discrete organization of the dorsal striatum cannot be applied to the NAc, and a “go”/“no-go” model does not capture the complexity of this system. The “direct” and “indirect” pathways are not distinctly organized in the NAc, as NAc D1 MSNs project to both the pallidum and tegmentum (Kupchik et al., 2015). These “direct and indirect” D1 MSN subpopulations are functionally distinct and can exert opposing effects on reward and aversion (Liu et al., 2022). Evidence for anatomically-distinct subpopulations exist across dorsal-ventral and medial-lateral gradients, and subpopulations have been distinguished between different afferent- and efferent- projections (Soares-Cunha et al., 2018; Yao et al., 2021; Zhou et al., 2022). In addition to anatomical differentiations, recent work by, Chen et al. identified eight genetically and functionally distinct D1 and D2 MSN subpopulations (Chen et al., 2021). This diversity aligns with imaging data using a gradient index (GRIN) lens to record calcium activity in individual neurons. Dominguez et al. characterized subpopulations of D1 and D2 MSNs that dynamically encode both unconditioned stimuli and associative learning (Domingues et al., 2025). These findings necessitate further characterization of NAc MSN diversity to fully appreciate their role in behavioral regulation.

### Limitations to mechanistic interpretation

Our interpretation is based on regulation by post-synaptic DRD2 receptors, but presynaptic DRD2 receptors are also present on the terminals of dopaminergic (and other) afferents. While our pharmacological approach does not rule out the contribution of presynaptic DRD2s, prior work by Hikida et al. (Hikida et al., 2013) found that quinpirole impaired inhibitory avoidance learning, and that mice that lack postsynaptic DRD2 receptors showed significant impairments in aversion learning. This demonstrates the necessity of postsynaptic DRD2 receptors in an aversion-learning task and suggests that the present findings may also reflect aversion-learning deficits that are caused by quinpirole activity at the postsynaptic DRD2 receptors. Due to several methodological differences, additional studies would be necessary to determine if both results reflect a common mechanism. It is also important to note that both cholinergic neurons and astrocytes in the striatum express DRD2s and have been shown to modulate terminal DA release and downstream MSN activity (Corkrum and Araque, 2021; Stedehouder et al., 2024). Quinpirole administration is highly likely to affect this process which could contribute to the observed effects.

However, the functional output of this intra-accumbens modulation ultimately occurs at the projection neurons to modify downstream motor action plans and alter behavior. This final common pathway is also being modulated by the increased occupancy of DRD2 receptors by quinpirole. While the present pharmacological manipulation cannot precisely identify the mechanism, the goal of the present study was to ask the more basic question of whether escape behavior would be impaired if an aversive outcome failed to reduce dopamine signaling at DRD2 receptors expressed in the NAc core. In other words, does the acquisition of escape behavior require the aversion-induced reduction of dopamine at the NAc DRD2? In support of the importance of the MSN being an essential locus of the action of DRD2 activation, both chemogenetic and optogenetic manipulations have been used to isolate the effects of post-synaptic MSN activity in aversion-related behaviors including transient punishment (Cole et al., 2018; Lobo et al., 2010), risk aversion (Zalocusky et al., 2016), and social avoidance (Francis et al., 2015).

### Conclusion and future directions

The results of this study align with prior evidence that aversion-induced decreases in dopamine and DRD2 receptor activity, specifically, are an important regulator of aversion-learning. DRD2 receptor agonism attenuated escape, suggesting that this signal is necessary to maintain aversion-motivated behavior. These findings align with an opposing valence-encoding model of striatal signaling, but there is growing evidence that challenges this model. While it is clear that the complexity of D1 and D2 MSN activity in behavioral regulation is much greater than previously appreciated, clinical insights suggest that a valence-based model may provide therapeutic insight. Depleted dopamine due to Parkinson’s disease or experimental manipulation are associated with enhanced punishment learning but not reward learning, and this effect is reversed by DRD2-agonist medications (Frank et al., 2004; Robinson et al., 2010). A disruption to valence-free associative learning does not account for the absence of a reward-based learning effect observed in these clinical studies. Further, this demonstrates improved performance through enhanced aversion signaling, rather than reward-based motivation. In line with this, our lab has recently demonstrated that aversion-induced decreases in dopamine can precede both increased drug taking and escape in a negative reinforcement design (Grafelman et al., 2025). Importantly, increased performance does not directly imply reward-based motivation, and it is important to consider that a similar mechanism may also be engaged to increase motivated behavior during optogenetic or chemogenetic manipulations. To rigorously test if or how D1 and D2 MSNs encode valence, future studies should continue to characterize the heterogeneous cell subpopulations, and careful behavioral designs should be employed to isolate the effects of valence encoding from other factors including learning and motivation.

## Data Availability

The original contributions presented in the study are included in the article/supplementary material, further inquiries can be directed to the corresponding author.
